# Exosomes as biomarkers and therapy in type 2 diabetes mellitus and associated complications

**DOI:** 10.3389/fphys.2023.1241096

**Published:** 2023-09-08

**Authors:** Nihal Satyadev, Milagros I. Rivera, Nicole K. Nikolov, Adegbenro O. J. Fakoya

**Affiliations:** ^1^ Department of Neurology, Mayo Clinic Florida, Jacksonville, FL, United States; ^2^ University of Medicine and Health Sciences, Basseterre, St. Kitts and Nevis; ^3^ Ross University School of Medicine, Bridgetown, Barbados; ^4^ Louisiana State University Health Sciences Center, Shreveport, LA, United States

**Keywords:** exosomes, biomarkers, type 2 diabetes mellitus, diabetic nephropathy, diabetic neuropathy, diabetic retinopathy, diabetic cardiomyopathy

## Abstract

Type 2 diabetes mellitus (T2DM) is one of the most prevalent metabolic disorders worldwide. However, T2DM still remains underdiagnosed and undertreated resulting in poor quality of life and increased morbidity and mortality. Given this ongoing burden, researchers have attempted to locate new therapeutic targets as well as methodologies to identify the disease and its associated complications at an earlier stage. Several studies over the last few decades have identified exosomes, small extracellular vesicles that are released by cells, as pivotal contributors to the pathogenesis of T2DM and its complications. These discoveries suggest the possibility of novel detection and treatment methods. This review provides a comprehensive presentation of exosomes that hold potential as novel biomarkers and therapeutic targets. Additional focus is given to characterizing the role of exosomes in T2DM complications, including diabetic angiopathy, diabetic cardiomyopathy, diabetic nephropathy, diabetic peripheral neuropathy, diabetic retinopathy, and diabetic wound healing. This study reveals that the utilization of exosomes as diagnostic markers and therapies is a realistic possibility for both T2DM and its complications. However, the majority of the current research is limited to animal models, warranting further investigation of exosomes in clinical trials. This review represents the most extensive and up-to-date exploration of exosomes in relation to T2DM and its complications.

## 1 Introduction

Type 2 diabetes mellitus (T2DM) is one of the most common metabolic disorders in adults, and its prevalence is increasing proportionally to the epidemic of obesity (Dunstan et al., 2002). It is estimated that 527 million people worldwide have T2DM, and that it is the ninth leading cause of death (Dunstan et al., 2002; Zheng et al., 2018; Sun et al., 2022). T2DM has alarmingly increased in its incidence in the United States and other western countries, placing a significant burden on families and caregivers. In addition, its growing costs for treatment and management threaten healthcare systems at large (Ceriello et al., 2020). At the cellular level, the pathophysiology of T2DM involves impaired insulin secretion and insulin resistance. While this pathophysiology at large increases mortality, it also presents with numerous chronic complications, including diabetic angiopathy (DA), diabetic cardiomyopathy (DCM), diabetic nephropathy (DN), diabetic peripheral neuropathy (DPN), diabetic retinopathy (DR), and diabetic wound healing (DWH). Given the significant burden, both to individuals and healthcare systems, several efforts have been made to identify novel biomarkers and therapeutics that would elucidate improved diagnosis and treatment of T2DM. One such effort has been through the discovery and study of exosomes, small vesicles that contain soluble and membrane-bound proteins, lipids, messenger RNA (mRNA), and DNA. Exosomes are involved in the autocrine, paracrine, and endocrine communication between cells. Over the past 10 years, these cellular communication packets have been implicated in the pathogenesis of T2DM, suggesting their utility in earlier recognition or novel diagnostic modality (i.e., via urine) (Chang and Wang, 2019). Moreover, given that the exosomes have been noted in various biochemical pathways implicated in T2DM, they could further serve directly as therapeutics for the disease. Furthermore, work in many animal studies have identified the role of exosomes in the development of the previously listed diabetic complications. As clinical trials are yet to make significant progress in this field of study, this review serves to describe exosomes and T2DM in detail and present a comprehensive assessment of exosome-related studies in T2DM and its associated complications. The findings in this review can be utilized to holistically understand the landscape of exosomal T2DM as a precursor to the development of novel studies in both animal models and clinical trials.

## 2 Exosomes

**FIGURE 1:**
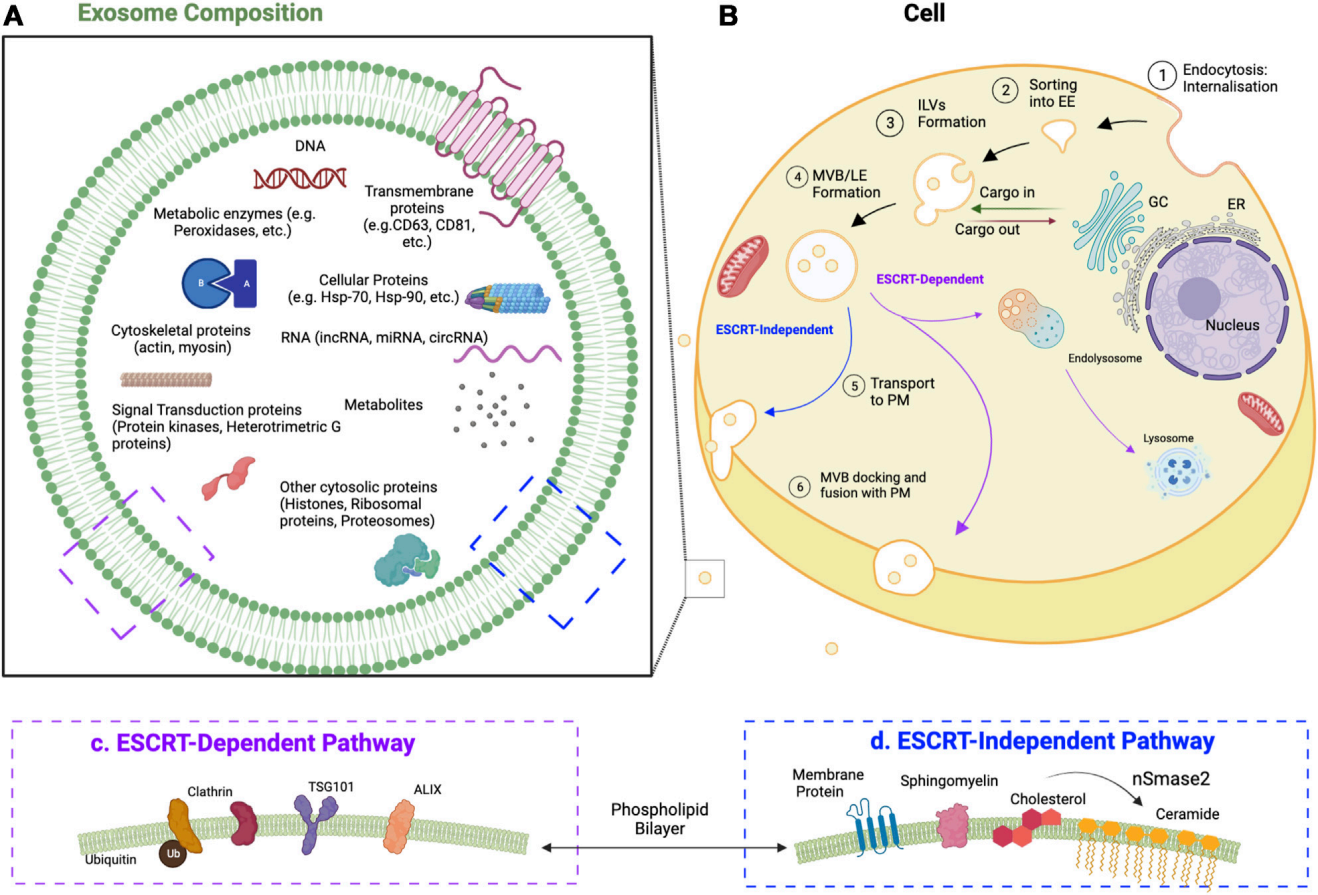
Development of exosomes and their components. **(A)** Exosome composition, common components of exosomes and their membranes. CD, cluster of differentiation; circRNA, circular RNA; Hsp, heat shock protein; lncRNA, long noncoding RNA; miRNA, microRNA **(B)** Cell, the developmental process of exosomes. Exosomes start as early endosomes (EE) and are trafficked to become multivesicular bodies (MVBs) (i.e., late endosomes; LE) containing intraluminal vesicles (ILVs). When these ILVs bud out of the plasma membrane (PM) outside of the cell, they are known as exosomes. **(C)** ESCRT-dependent exosomal markers. ALIX, apoptosis-linked gene 2-interacting protein X; TSG101, tumor susceptibility gene 101 protein **(D)** ESCRT-independent exosomal markers. nSmase2, neutral sphingomyelinase 2.

Exosomes are extracellular vesicles (EVs) that are released by cells when multivesicular bodies fuse with the plasma membrane. This fusion liberates the contents within the multivesicular bodies (MVBs), which are known as intraluminal vesicles (ILVs), into the extracellular environment (Edgar, 2016). Exosomes (i.e., released ILVs) have a diameter between 40 and 100 nm and a characteristic cup-shaped morphology that can be observed via electron microscopy (Conde-Vancells et al., 2008). Exosomes first start as early endosomes, which then bud inward to generate ILVs and become endosomes. Various contents (e.g., proteins, lipids, nucleic acids) are packaged into these ILVs by machinery that may or may not involve the endosomal sorting complex required for transport (ESCRT). Through this process, endosomes mature into MVBs. The ESCRT-dependent pathway targets MVBs to the plasma membrane via mechanisms that are well understood (Williams and Urbé, 2007; Hurley, 2008). Processes that do not involve the ESCRT (i.e., non-ESCRT) are poorly characterized but are suggested to involve ceramide and the subsequent generation of raft-lipids, which motivate the MVBs to reach the plasma membrane rather than to participate in lysosomal fusion (Trajkovic et al., 2008). Exosomes have several specific cytoplasmic markers regardless of the cell in which they are generated. For example, exosomes contain proteins involved in membrane transport and fusion (e.g., Rab GTPases, Annexins, flotillin), heat shock cognate proteins (e.g., hsc70, hsc90), integrins, and tetraspanins (e.g., CD63, CD9, CD81, CD82). In addition, exosomes generated through the ESCRT pathway contain apoptosis-linked gene 2-interacting protein X (ALIX) and tumor susceptibility gene 101 protein (TSG101) (Figure 1) (Wubbolts et al., 2003; Simons and Raposo, 2009).

Exosomes were first described in 1983 by Harding et al. when the group was attempting to understand the maturation of reticulocytes to erythrocytes (Harding et al., 1983). Using pulse-chase experiments and electron microscopy, the group was able to identify late endosomes with MVBs that could fuse with the plasma membrane, releasing contents extracellularly rather than fusing with lysosomes (Harding et al., 1983). A few years later, the term “exosomes” was coined (Johnstone et al., 1987). However, their relevance was minimal for several decades until nearly the turn of the century when it was discovered that exosomes harbor functional major histocompatibility complex (MHC)-peptide complexes that induced immune responses *in-vivo* (Zitvogel et al., 1998). Subsequently, exosomal content was analyzed, suggesting that exosomes contained proteins, lipids, mRNA, microRNA (miRNA), long non-coding RNA (lncRNA), circular RNA (circRNA), and DNA (Théry et al., 1999; Théry et al., 2001; Valadi et al., 2007; Ronquist et al., 2012). miRNA is known to primarily regulate mRNA, and both lncRNA and circRNA primarily regulate miRNA (Morris and Mattick, 2014). At around the same time as the discovery of exosomal components, it was noted that exosomes are mediators of intercellular communication (Théry et al., 2002). All of these findings together, along with a later finding that most exosomes actually do not contain MHCs, suggested to the scientific community that exosomes could play an integral role in therapeutic medicine (Kim et al., 2016). The concept of utilizing exosomes, especially those derived from stem cells, without the original cells themselves as a form of therapy is known as “cell-free” therapy (Phinney and Pittenger, 2017).

Although exosomes are defined specifically above, they cannot always be studied in isolation. This is because exosomal purification is done by ultrafiltration, which can, at best, separate vesicles between 50 and 1,000 nm in diameter (Cheruvanky et al., 2007). The inclusion of larger-than-exosomal products has been assigned various terms (e.g., microsomes, ectosomes), which, together with exosomes, form the umbrella term of extracellular vesicles (Stein and Luzio, 1991; Théry, 2011). While this limitation is respected, the findings in this review include work primarily from papers that confirm exosomal markers (e.g., ALIX, TSG101, CD9, *etc.*).

Exosomes are known to be involved in several physiological and pathological functions. To provide one physiological example, in the central nervous system, exosomes are known to have an important role in myelin sheath maturation, neuronal activity levels, and axonal regeneration (Court and Alvarez, 2005; Bakhti et al., 2011; Frühbeis et al., 2013). Pathologically, exosomes have been implicated in cancer, heart disease, neurodegenerative disorders, and even psychiatric conditions (Liu W. et al., 2019; Saeedi et al., 2019; Zheng et al., 2020; Zhang et al., 2021; Zhou et al., 2021). Having explored an introductory explanation of exosomes, the next section explores the fundamentals of T2DM.

## 3 Type 2 diabetes mellitus: insulin production and pathophysiology in brief

### 3.1 Insulin production

The pancreatic β-cells are responsible for insulin production. Insulin begins as pre-proinsulin and is subsequently modified and cleaved in the endoplasmic reticulum and Golgi apparatus, respectively, to yield insulin and C-peptide (Halban, 1994; Fu et al., 2013; Bunney et al., 2017). Insulin is then stored in intracellular granules until the β-cells receive a signal to release insulin. This signal is commonly triggered by glucose entering the cell via glucose transporter 2 (GLUT2); however, it can also be triggered by amino acids, fatty acids, and hormones (Boland et al., 2017). Once glucose enters the cells, the ATP to ADP ratio rises, triggering the closure of ATP-dependent potassium channels, membrane depolarization, and calcium intracellular influx, which ultimately triggers insulin exocytosis (Seino et al., 2011; Boland et al., 2017). This process can also be triggered by cyclic AMP and is amplified through ryanodine receptor activation (Islam, 2002; Cuíñas et al., 2016).

### 3.2 Pathogenesis of type 2 diabetes mellitus

T2DM is characterized by hyperglycemia resulting from impaired insulin secretion, insulin resistance, or a combination of both (Roden and Shulman, 2019). Insulin regulates glucose metabolism and maintains normal blood sugar levels in the human body by promoting glucose uptake and utilization in peripheral tissues (Stout, 1979). Insulin levels must accurately meet the metabolic demand to avoid hypoglycemia or hyperglycemia. For this reason, the synthesis and release of insulin and the ensuing insulin response in tissues are tightly regulated (Roden and Shulman, 2019). Defects in this regulation can lead to a metabolic imbalance that leads to the pathogenesis of T2DM. Unlike the β-cells that are affected in type 1 diabetes mellitus through autoimmunity, impairment of insulin secretion in T2DM occurs in a multifactorial, non-autoimmune process (Halban et al., 2014; Kawasaki, 2014).

#### 3.2.1 Genetic influence in the pathogenesis of insulin secretion

Of the over 60 genes that have been associated with T2DM, the large majority are involved in the insulin pathway with β-cells (Voight et al., 2010; Rosengren et al., 2012). Although some of these variants are known to incur high risk, one’s genetic profile generally does not significantly indicate diabetic risk (Manolio et al., 2009; Albrechtsen et al., 2013). Epigenetics, the modification of gene expression, however, does play a more significant role in the pathology’s development. For example, several *in-vitro* and *in-vivo* studies have shown DNA methylation to be key in down-regulating genes that are needed for insulin production (Volkmar et al., 2012; Yang et al., 2012; Dayeh et al., 2013). Epigenetic modification by histone deacetylases has also been shown to lead to cytokine-mediated β-cell damage (Christensen et al., 2011). Of most specific interest to this work is the upregulation and downregulation of several miRNAs that have been linked to the development of (and alternatively, the protection against) T2DM (Poy et al., 2004; Poy et al., 2009; Guay and Regazzi, 2013). Interestingly, miRNAs have been shown to also bind to mRNAs coding for DNA methylation proteins, suggesting they have a multifactorial epigenetic role (Breving and Esquela-Kerscher, 2010). Given that miRNAs are a key component within exosomes, their effects are further detailed later in this paper.

#### 3.2.2 Other regulators of insulin secretion

β-cell insulin secretion is affected by several sources of stress, including inflammation, amyloid build-up, endoplasmic reticulum overload, and reactive oxidative species formation. In regards to inflammation, studies have revealed that elevated glucose and free fatty acids impair insulin production through the ultimate downregulation of the anti-inflammatory interleukin-1 (IL-1) receptor antagonist and upregulation of pro-inflammatory IL-1β and FAS (Donath and Shoelson, 2011). These proinflammatory proteins lead to decreased insulin secretion and β-cell apoptosis, respectively (Maedler et al., 2002; Schumann et al., 2007). Islet amyloid polypeptide buildup is seen in nearly 90% of T2DM cases on autopsy and is known to correlate with disease severity (Westermark, 1994; Kahn et al., 1999). Although the mechanism of amyloid buildup is debated, it is known that the buildup induces β-cell apoptosis and further inflammation (Donath and Shoelson, 2011; Raleigh et al., 2017). Endoplasmic reticulum stress is generated when high levels of insulin are required, such as due to hyperglycemia, which causes the protein to become misfolded. Although several mechanisms exist to mitigate this misfolding, chronic endoplasmic reticulum stress leads to β-cell apoptosis (Harding and Ron, 2002; Laybutt et al., 2007). Finally, reactive oxygen species, which are known to form in β-cells as a result of both acute and chronic hyperglycemia, function in a multifactorial way, including furthering endoplasmic reticulum stress, inhibiting calcium mobilization, and ultimately inducing apoptosis (Volpe et al., 2018).

#### 3.2.3 Insulin resistance

Insulin resistance broadly refers to a reduced metabolic response to insulin, which, at a systemic level, leads to a diminished lowering of blood glucose for the same amount of circulating insulin. Beyond T2DM, insulin resistance is seen in most metabolic disorders, such as obesity, dyslipidemia, and metabolic syndrome (Samuel and Shulman, 2016). Studies of insulin resistance have shown that the relationship between insulin sensitivity and insulin release is non-linear, with a shape representative of the positive quadrant of the cosecant hyperbolic curve. In other words, as the sensitivity decreases below a certain threshold, the amount of insulin needed grows exponentially (Kahn et al., 2006). Insulin resistance, especially in relation to its effect on tissues, is known to precede T2DM by 10–15 years (Freeman and Pennings, 2022). Amongst these tissues, the three most critical to the development of T2DM are skeletal muscle, adipose tissue, and liver (DeFronzo and Tripathy, 2009; Galicia-Garcia et al., 2020). In physiological conditions, skeletal muscle is known to absorb approximately 80% of glucose (Thiebaud et al., 1982). Although the precise underpinnings of the development of skeletal muscle insulin resistance are still under investigation, several proposed mechanisms act by reducing GLUT4 translocation to the membrane of the skeletal muscle cells (Petersen and Shulman, 2002). One consideration is the upregulation of protein-tyrosine phosphatase 1B (PTP1B), an enzyme known to counteract the phosphorylation of insulin receptor substrate-1 (IRS-1), which otherwise signals membrane translocation of GLUT4 (Yip et al., 2010). Inflammation, through the IKKβ/NF-κB and JNK pathways, is also suggested to be involved in the development of insulin resistance. Both of these pathways lead to disruption of IRS-1 function (de Luca and Olefsky, 2008). Several cytokines have been associated with this process, but crucially, adiponectin is known to be protective against insulin resistance (Fantuzzi, 2005). Beyond inflammation and PTP1B, other proposed mechanisms include mitochondrial dysfunction, endoplasmic reticulum stress, GLUT4 mutations, and reduction in IRS-1 binding capacity (Yaribeygi et al., 2019).

## 4 Exosomes in type 2 diabetes mellitus

Several exosomes have been shown to contribute to insulin resistance. Adipocytes, for example, have been shown to release exosomes carrying sonic hedgehog protein, which in turn induces proinflammatory activation of monocytes, ultimately leading to insulin resistance (Song et al., 2018). In another study, exosome-like vesicles from murine adipose tissue were shown to similarly induce insulin resistance by motivating the upregulation of tumor necrosis factor alpha (TNF-α) and IL-6 (Deng et al., 2009). Conversely, exosomes can also play a protective role, as in the case of β-cells releasing exosomal neutral ceramidase to infer paracrine protection against free fatty-acid-induced apoptosis (Tang et al., 2017).

Although exosomal proteins have been described to be key regulators of T2DM, the majority of the focus of exosomal effects has been on their miRNA components. MiRNAs are small noncoding RNAs, generally 19–22 nucleotides in length, that function by binding mRNA to induce their decomposition or inhibit their translation (Bartel, 2009). Several studies have confirmed their role in the modulation of metabolic disorders, including the regulation of lipid and glucose metabolism, insulin secretion, and gluconeogenesis (Foley and O’Neill, 2012; Jiang et al., 2013; Vickers et al., 2014; Brennan et al., 2017; Massart et al., 2017). In T2DM specifically, the PI3K-Akt pathway, which is responsible for the translocation of the GLUT4 transporter to the plasma membrane, was initially shown to be regulated by exosomes in an *in vitro* study using pancreatic cancer cells (Wang et al., 2017). Human studies found the pathway was downregulated by exosomal miR-20b-5p, which was shown to be significantly increased in T2DM patients compared to healthy controls (Katayama et al., 2019). Studies of other pathways implicated in T2DM in diabetic mice models found that exosomal miR-155 induced glucose through the targeting of PPARγ and that healthy mice could develop T2DM through the introduction of miR-122, miR-192, miR-27a-3p, and miR-27b-3p; which collectively downregulated PPARα (Ying et al., 2017; Castaño et al., 2018). Such findings suggest that exosomes could be the underlying modulators that progress a healthy individual to develop T2DM (Table 1).

**TABLE 1:** The role of exosomal components in Type 2 diabetes mellitus and related models *in-vivo*. ap2, Fatty acid-binding protein 4; lncRNA, long non-coding RNA; miR, microRNA; NR, not reported; PEPCK, phosphoenolpyruvate carboxykinase; p-STAT3, phosphorylated signal transducer and activator of transcription 3; RBP4, retinol binding protein 4.

Exosomal component	Source	Species	Primary purpose	Location	Association with T2DM	References
miR-16	Skeletal muscle	Mouse	Therapeutic	Serum	Increased	Jalabert et al. (2016)
ap2	Adipocyte	Mouse	Therapeutic	Serum	Increased	Ertunc et al. (2015)
miR-15a	Pancreatic β-cells	Human	Diagnostic	Serum	Increased	Kamalden et al. (2017)
lncRNA-p3134	Pancreatic β-cells	Human	Diagnostic	Serum	Increased	Ruan et al. (2018)
miR-20b-5p	NR	Human	Pathologic	Serum	Increased	Katayama et al. (2019)
miR-122, miR-192, miR-27a-3p, miR-27b-3p	NR	Mouse	Pathologic	Serum	Increased	Castaño et al. (2018)
miR-155	Adipose tissue macrophages	Mouse	Pathologic	NR	Increased	Ying et al. (2017)
miR-29b-3p	Bone marrow mesenchymal stem cells	Mouse	Pathologic	Serum	Increased	Su et al. (2019)
miR-375-3p	Pancreatic β-cells	Human	Diagnostic	Serum	Increased	Fu et al. (2018)
p-STAT3	Adipose-derived stem cells	Mouse	Therapeutic	Serum	Increased	Zhao et al. (2018)
RBP4	Adipocytes	Mouse	Pathogenic	NR	Increased	Deng et al. (2009)
miR-29a	Adipose tissue macrophages	Mouse	Pathogenic	Serum	Increased	Liu et al. (2019a)
miR-27a	Adipocytes	Mouse	Pathogenic	Serum	Increased	Yu et al. (2018)
miR-141-3p	Adipocytes	Mouse	Pathogenic	Serum	Decreased	Dang et al. (2019)
miR-130a-3p	Hepatocytes	Mouse	Therapeutic	Serum	Decreased	Wu et al. (2020b)
miR-26a	β-cells	Mouse	Therapeutic	Serum	Decreased	Xu et al. (2020)
PEPCK	NR	Human/Rat	Diagnostic	Urine	Increased	Sharma et al. (2020)
let-7b, miR-144-5p, miR-34a, and miR-532-5p	NR	Human	Diagnostic	Serum	Increased	Jones et al. (2017)
miR-20b-5p	NR	Human	Diagnostic	Serum	Increased	Katayama et al. (2019)
miR-491-5p, miR-1307-3p, miR-298	NR	Human	Diagnostic	Serum	Increased	Sidorkiewicz et al. (2020)

Beyond implications in pathogenesis, several exosomal miRNAs, in serum and urine, have been found to be correlated, either inversely or directly, to the presence of T2DM (Table 1). These findings suggest that exosomes, particularly due to their miRNA components, could serve as excellent biomarkers to determine the progression to T2DM pre-clinically. Beyond the role of exosomes in T2DM at large, several studies have identified exosomes as possible biomarkers and therapeutics in the disease’s complications.

## 5 Exosomes in complications of type 2 diabetes mellitus

### 5.1 Diabetic angiopathy

DA is a common underlying pathology in diabetic patients. It is subdivided into macroangiopathy, which affects larger arteries, and microangiopathy, which includes endothelial damage to the vessels between primary arterioles and venules and is largely associated with chronic T2DM complications. The pathophysiology of DA is not well understood; however, it is believed to involve several pathways that are affected by reactive oxygen species (Hammes, 2003). Of importance, is the involvement of angiogenesis in DA as its unequal balance (i.e., of pro-angiogenic and anti-angiogenic factors) is known to be pathogenic for several T2DM complications (Xu et al., 2012).While the majority of exosomal studies focused on organ-specific diabetic complications (e.g., DCM), a few focused on DA itself. These studies specifically focused on the role of exosomes in vascular smooth muscle cells (VSMCs). VSMCs surround the endothelial cells of blood vessels and are known to contribute to oxidative damage in T2DM (Hattori et al., 2000; Inoguchi et al., 2000).

Wu et al. was able to identify that *in-vitro* VSMCs in high-glucose conditions contained higher levels of carnitine palmitoyltransferase 1 (CPT1A), and that this enzyme contributed to inflammation in the blood vessels as measured by levels of several proinflammatory markers (e.g., TNF-α, IL-6, *etc.*). Furthermore, the group identified exosomal miR-324-5p to be an inhibitor of CPT1A and then confirmed the findings by comparing VSMCs harvested from patients with and without T2DM (Wu G. et al., 2020). Using the same methodologies, another group was able to identify another DA pathway that involved downregulation of lncRNA UCA1 which led to upregulation of miR-582-5p and ultimately pathogenesis (Yang and Han, 2020). Interestingly, the findings from both these studies found levels of the studied RNAs and enzymes to be modified both within serum exosomes and within VSMCs themselves. This suggests that pathway modification due to exosomal signaling may occur before, during, or after exosomal transport (Wu G. et al., 2020; Yang and Han, 2020).

The studies above highlight exosomes in microangiopathic DA. Similarly, the other complications from T2DM listed in the remaining sections are associated with microangiopathic disease. Complications related to macroangiopathy, such as stroke and atherosclerosis, were omitted from this review because the underlying contributors are generally considered not to be isolated to T2DM alone (i.e., overlap with hypertension) (Verhagen and Visseren, 2011; Frostegård, 2013; Murphy and Werring, 2020). However, it should be noted that the role of exosomes in these pathologies is an active area of research (Venkat et al., 2018; Wang et al., 2018; Guo et al., 2022)**.**

### 5.2 Diabetic cardiomyopathy

DCM is the occurrence of heart failure in diabetic patients that cannot be attributed to hypertension or coronary artery disease (Gilbert and Krum, 2015). Nearly 50% of T2DM patients with no cardiac symptoms have been reported to have diastolic dysfunction, suggesting that DCM may be severely underdiagnosed (Patil et al., 2011). DCM pathology is multifactorial, with groups reporting the involvement of factors such as oxidative stress, inflammation, the lack of myocardial angiogenesis, lipid accumulation, and fibrosis (Bugger and Abel, 2014). Despite this, the true pathophysiological underpinnings of DCM are yet to be ascertained. For a while, however, it has been known that exosomes play an important role in the processes of myocardial repair and remodeling (Ibrahim et al., 2014; Sahoo and Losordo, 2014; Waldenström and Ronquist, 2014). And more recently, greater focus has since been provided to their role, specifically in models of DCM.

Building on their group’s earlier findings that genetic deletion of matrix metallopeptidase 9 (MMP9) improves cardiac contractility and repair in diabetic patients, the Tyagi group aimed to assess whether cardiac exosomes were regulating MMP9 (Moshal et al., 2008; Mishra et al., 2012; Chaturvedi et al., 2015). Using a db/db diabetic mice model (i.e., downregulation of the leptin receptor), the group screened for four miRNAs that were sequentially similar to the 3′ region of the MMP9. They hypothesized that exercise would upregulate the production of miRNAs which would suggest inhibitory regulation of MMP9 expression. They ultimately found that one of those miRNAs, miR-455, was significantly upregulated in exercise and that its presence significantly downregulated MMP9 (Chaturvedi et al., 2015). Taken together with the group’s earlier findings, this provided the first evidence that the benefits of exercise in DCM could be measured through miRNA biomarkers (Moshal et al., 2008; Mishra et al., 2012; Chaturvedi et al., 2015).

Since then, other groups have discovered additional biomarkers for this pathology. De Gonzalo-Calvo et al. tested a few miRNAs that were known to previously have been signs of myocardial ischemia to assess their correlation with DCM (Bostjancic et al., 2010; de Gonzalo-Calvo et al., 2017). The group assessed miRNAs not only *in-vitro* and *in-vivo* but also in humans with DCM without other diabetic complications. Their findings suggest that serum miR-1 and miR-133a are strongly correlated with the presence of myocardial steatosis, as well as some functional heart indicators such as E/Ea ratio. Furthermore, the group was able to create a multivariable logistic regression model that used miR-1 and miR-133a as well as five demographic and basic lab values to generate a predictive c-statistic of 0.88 (95%CI: 0.79–0.97). This group was the first to assess miRNA biomarkers in humans for DCM, and their findings suggest realistic clinical applicability of this biomarker. However, their findings are not without limitations, most notably that 14 to 22 percent of the patients had serum levels that were below the limit of detection, and that only male patients were studied (de Gonzalo-Calvo et al., 2017). Veitch et al. also looked into miRNAs that could serve as biomarkers. However, this group’s findings looked at EVs as a whole and not just exosomes. Still, it was notable that they isolated miR-30d-5p and miR-30e-5p as serum markers in both mice and rats that correlated with cardiac microvascular endothelial dysfunction and preceded diastolic dysfunction. Interestingly, these miRNAs were noted only to be upregulated in the left ventricle when compared with tissue from six other organs. This finding suggests that highly specific miRNA biomarkers for DCM may exist that precede symptomology. Unsurprisingly, the group also discovered this miRNA family to be involved in key *in-vitro* and *in-vivo* pathways that have been suggested to be involved in the DCM pathogenesis (Veitch et al., 2022).

Beyond the findings from these two in-depth studies, miRNA families 1, 34, 143, 145, 150, 181, 214, 223, 320, 373, 374, 449, and 761 have been hypothesized to be involved in the pathogenesis of DCM. Most of these families, however, either have not had rigorous studies as to their potential as biomarkers or have not been confirmed to be found in exosomes (Nandi and Mishra, 2018). Several other exosomal components, however, have also been noted to be generally cardio-toxic. For example, miR-21 and miR-146a, which are released by cardiac fibroblasts; miR-320, which are released by diabetic cardiomyocytes; and NADPH, nitro oxid synthetases, and protein disulfide isomerase, which are released by platelets have all been shown to be toxic to cardiomyocytes and/or promote cardiomyocyte hypertrophy (Janiszewski et al., 2004; Azevedo et al., 2007; Halkein et al., 2013; Bang et al., 2014; Indolfi and Curcio, 2014; Wang et al., 2014). This suggests a myriad of possible therapeutic targets for DCM (Ailawadi et al., 2015; Nandi and Mishra, 2018).

One of the groups assessing these deleterious effects noticed that diabetic cardiomyocytes produced exosomes with downregulated heat shock protein 20 (Hsp-20) (Wang et al., 2014; Ailawadi et al., 2015). The group subsequently hypothesized that upregulation of exosomal Hsp-20 could mitigate the deterioration of cardiac function in DCM. The group first confirmed that upregulation of Hsp-20 conferred cardioprotective effects using a streptozotocin-injected Hsp-20 transgenic mouse model. Importantly, the group demonstrated colocalization of Hsp-20 with TSG101, suggesting that Hsp-20 is housed in exosomes released from cardiomyocytes. Further *in-vivo* and *in-vitro* analysis demonstrated that these exosomes also included protective proteins (i.e., p-Akt, superoxide dismutase 1, and survivin). That same analysis also confirmed that upregulation of these exosomes leads to reduced oxidative stress, decreased fibrosis, and increased angiogenesis, all of which suggest mitigation of DCM (Wang et al., 2016). Another group identified a separate cardioprotective exosomal heat shock protein, Hsp-70 (Vicencio et al., 2015). Interestingly, the pathway the group identified to be activated by Hsp-70 (i.e., ERK1/2) was no longer activated in a diabetic rat model in spite of them containing exosomal Hsp-70. However, when exosomes were isolated control rats and injected into diabetic rats, cardioprotective pathways were again activated. These findings suggest that exosomal Hsp-70 is likely glycated during diabetic onset and loses its potential to activate cardioprotective ERK1/2 (Davidson et al., 2018).

Beyond Wang et al., a few other groups have also made key findings about exosomal therapeutic targets for DCM. Hu et al., having previously proposed mammalian sterile 20-like kinase 1 (Mst1) to be a key regulatory protein of autophagy and apoptosis in DCM, attempted to characterize whether or not the protein was involved in the pathology as an exosomal component (Lin et al., 2016; Zhang et al., 2016; Zhang M. et al., 2017; Hu et al., 2018). In a mouse model, the group was able to show that Mst1 is created in cardiac endothelial cells and subsequently transported to cardiomyocytes through exosomes. Further *in-vitro* work suggested that endothelial Mst1 inhibits physiologic autophagy, promotes apoptosis, and suppresses glucose metabolism in cardiomyocytes (Hu et al., 2018). Zhang et al. attempted to assess the therapeutic effects of exosomes as a whole by extracting them from cultured human umbilical cord mesenchymal cells. Shockingly and excitingly, injecting these exosomes into diabetic rats with established DCM was able to reverse the pathology, as measured by histology and functional heart metrics. The group further demonstrated that the effects were mediated through the upregulation of the AMPK-ULK1 signaling pathway, which in turn inhibits pathological autophagy (Zhang et al., 2022).

Ultimately, the pathogenesis of DCM is complicated and not fully understood. However, several groups have demonstrated that its pathogenesis intimately involves a myriad of exosomes and exosomal components (Table 2). Although much of the research done thus far is limited to *in vitro* models and animal studies, exosomes and exosomal components are appearing promising as biomarkers and therapeutics for DCM.

**TABLE 2:** The role of exosomal components in diabetic cardiomyopathy *in-vivo*. DCM, diabetic cardiomyopathy; ERK, extracellular signal-regulated kinase; GLUT4, glucose transporter type 4; HSP, heat shock protein; MiR, miRNA, microRNA; MSC, mesenchymal stem cell; Mst1, mammalian sterile 20-like kinase 1; p-Akt, phosphorylated protein kinase B; SOD1, superoxide dismutase 1.

Primary purpose	Exosomal component	Species	Location	Association with T2DM complication	Potential implications	References
Diagnostic	miR-1, miR-133a	Mouse, Human	Serum	Increased	Correlated with the progression and presence of DCM	de Gonzalo-Calvo et al. (2017)
Diagnostic	miR-30d-5p, miR-30e-5p	Mouse, Rat	Serum	Increased	Correlated with microvascular changes in DCM and precede diastolic dysfunction. Hence, these may be the earliest miRNAs increased in the development of DCM.	Veitch et al. (2022)
Therapeutic	miR-455, miR-29b	Mouse	Serum	Decreased	Implicated in exercise-related protection of the diabetic heart from developing DCM	Chaturvedi et al. (2015)
Therapeutic	Mst1	Mouse	Cardiomyocytes (paracrine)	Increased	Exosomal Mst1 is increased in mice with DCM and has been shown to disrupt GLUT4 membrane translocation	Hu et al. (2018)
Therapeutic	Hsp-20, p-Akt, SOD1, survivin	Mouse	Cardiomyocytes (paracrine)	Decreased	Hsp-20 transgenic model yielded cardioprotection against DCM and was noted to have exosomes with several cardioprotective components (i.e., p-Akt, SOD1, and survivin) and Hsp-20 itself	Wang et al. (2016)
Therapeutic	Hsp-70	Rat	Cardiomyocytes (paracrine)	Decreased	Hsp-70 activated cardioprotective ERK1/2 pathway, however, its function is lost in exosomes from diabetic rats. Cardioprotection can be restored using non-diabetic serum exosomes *in-vitro*	Davidson et al. (2018)

### 5.3 Diabetic nephropathy

DN is a complex microvascular complication that generally presents in patients ten to 20 years after diagnosis of T2DM (Young et al., 2003). More than 40% of patients with T2DM develop DN, making it the leading cause of end-stage renal disease in the Western world (Gnudi and Gentile, no date). It is characterized by glomerular basement membrane thickening, mesangial sclerosis, and endothelial dysfunction (Haraldsson et al., 2008). In addition, it is suggested that epithelial-mesenchymal transition may play a role in the development of diabetic nephropathy through the advancement of renal fibrosis (Yamaguchi et al., 2009). Although diabetic nephropathy itself is not the most common cause of death in T2DM, it increases the risk of cardiovascular complications, which are the leading cause of death in T2DM (Gross et al., 2005). Hence, early recognition and subsequent mitigation of diabetic nephropathy are crucial, and exosomes offer that possibility.

Before studies specifically focused on exosomes, one study focused on the potential of proteins within microvesicles. In this observational study, the group found microvesicle-bound dipeptidyl peptidase-4 (DPP-4) activity to be decreased in the serum. Conversely, it was found to be increased in the urine of T2DM patients with DN when compared to non-diabetic healthy controls (Sun et al., 2012). This positive correlation further confirms previous findings that suggested the direct relationship between DPP-4 activity and worsening DN (Ahrén, 2007; Sun et al., 2012). Hence, microvesicular DPP-4 could serve as a biomarker for the diagnosis and progression of DN.

After this, a few other studies identified exosomal proteins associated with DN (Raimondo et al., 2013; Zubiri et al., 2014). Raimondo et al. conducted a proteomic analysis of 286 proteins found in urinary exosomes from Zucker diabetic fatty rats and compared them to non-diabetic rats (Raimondo et al., 2013). The levels of only two enzymes were found to be significantly different: Xaa-Pro dipeptidase and major urinary protein-1 (MUP1). Xaa-Pro dipeptidase, an enzyme involved in the metabolism of proline-containing peptides and proteins, was increased, which could indicate higher metabolic activity or elevated renal protein turnover (i.e., the rate that proteins are synthesized, degraded, and replaced). Conversely, MUP1, a protein mainly produced in the liver and secreted into the urine, was decreased. This may suggest impaired renal function or reduction in the synthesis and secretion of MUP1 in DN. Another study by Zubiri et al. utilized the more reliable ExoQuick reagent-based isolation technique to isolate exosomes from urine (Zubiri et al., 2014). Here, three exosomal proteins in urine were found to be possible biomarkers in DN patients: upregulated ɑ-1-microglobulin/bikunin precursor (AMBP), upregulated lysine-specific methyltransferase 2C (MLL3) and downregulated voltage-dependent anion channel 1 (VDAC1) (Zubiri et al., 2014). AMBP is a glycoprotein involved in inflammation by inhibiting proteases, exerting antioxidant effects, modulating cytokine activity, and influencing immune cell function (Grewal et al., 2005). AMBP has also been reported to be increased in T2DM patients’ urine, in comparison to healthy individuals (Pisitkun et al., 2004; Gonzales et al., 2009; Li et al., 2011). MLL3 is a histone methyltransferase involved in gene regulation and chromatin remodeling (Hosseini and Minucci, 2017). MLL3 has also been linked to the activation of PPAR𝛾, which in turn controls glucose by degrading SOCS5, a negative regulator of JAK/STAT pathway, consequently regulating glucose (Corrales et al., 2018). Finally, VDAC1 is a protein that is primarily located in the outer mitochondrial membrane, where it plays a critical role in regulating the transport of ions and metabolites, particularly the exchange of ATP and ADP. Since mitochondrial dysfunction has been observed in DN, VDAC1 expression or activity could contribute to the onset and progression of this pathology (Pinti et al., 2019). Zubiri et al.’s findings of decreased urinary VDAC1 reinforce the association of DN with increased rate of cell death and renal fibrosis (Zubiri et al., 2014). More recent work by Wen et al. identified that mice with diabetes had reduced expression of exosomes at large by the tubular interstitial cells (Wen et al., 2020). More specifically, their *in-vitro* Nephroseq analysis revealed a potential role for the enolase 1 protein in the pathogenesis of the disease as it was differentially expressed in various areas within the diabetic kidney and between non-high glucose and high glucose treated renal cells (Martini et al., 2008; Wen et al., 2020). Tao et al. also conducted similar genetic network analysis but focused biomarker study in humans. The group’s work identified adipocyte enhancer binding protein 1 to have a strong ability to distinguish DN patients from healthy controls (AUC = 0.880) and from T2DM patients (AUC = 0.742) (Tao et al., 2021).

**FIGURE 2:**
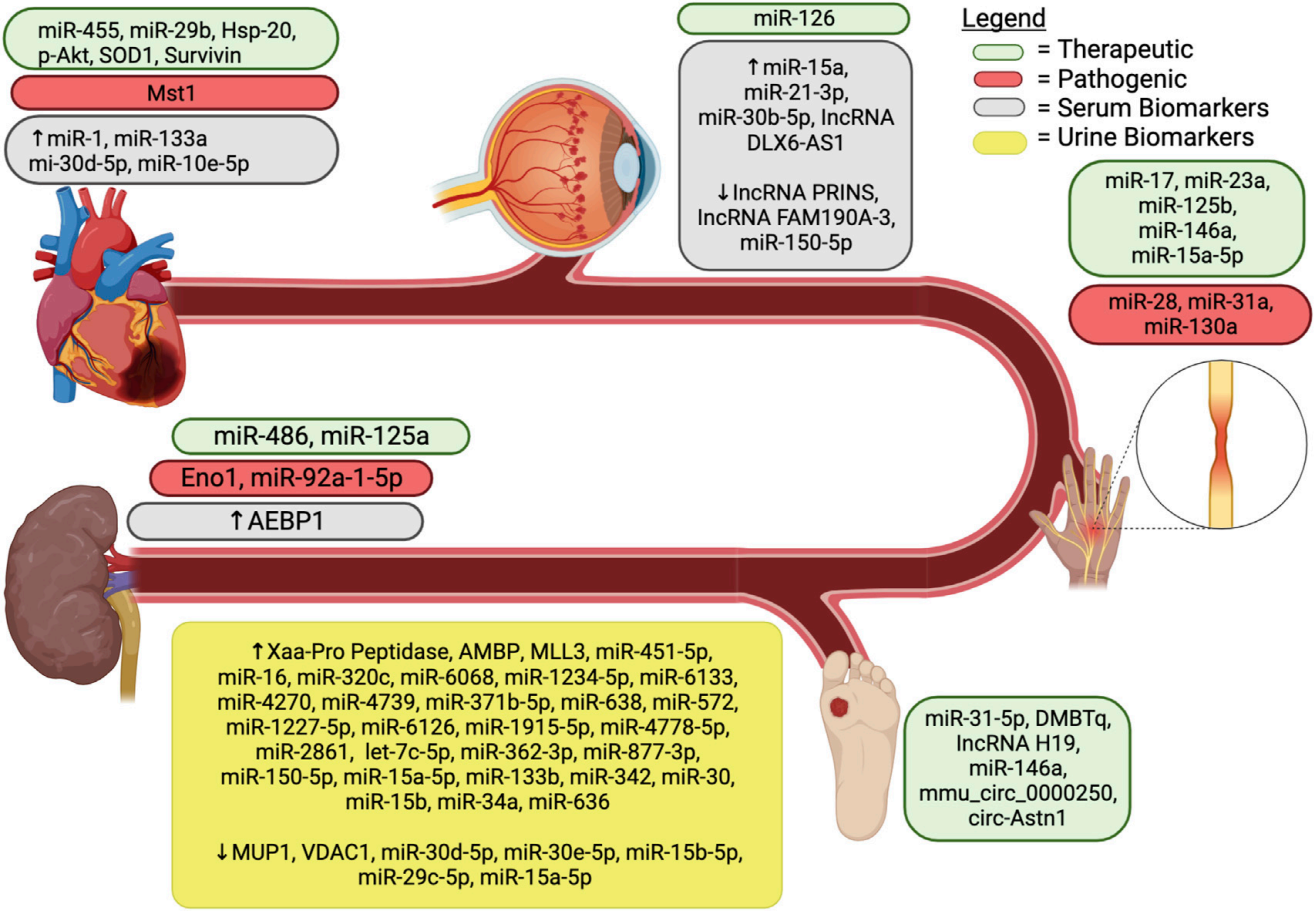
Exosomal components associated with diabetic complications. AEBP1, Adipocyte enhancer binding protein 1; AMBP, ɑ-1-microglobulin/bikunin precursor; DMBT1, deleted in malignant brain tumors 1; Eno1, enolase-1; MiR, microRNA; HSP, heat shock protein; lncRNA, long noncoding RNA; MSC, mesenchymal stem cell; MLL3, upregulated lysine-specific methyltransferase 2C; Mst1, mammalian sterile 20-like kinase 1; MUP1, major urinary protein 1; SOD1, superoxide dismutase 1; VDAC1, downregulated voltage-dependent anion channel 1.

Other exosomal cargo, such as miRNA, have also shown potential as biomarkers. Early work by Lv et al. identified reduced exosomal miR-29 and miR-200 families as biomarkers for chronic kidney disease. The group was able to create various models that predicted the extent of renal fibrosis with high AUCs (i.e., 0.9 and above) (Lv et al., 2013). More recent work focused on DN, was able to create a multivariable model using urine exosomal miR-126, miR-146, miR-155, as well as other paraclinical data, to predict between patients with T2DM with and without DN while maintaining high sensitivity (0.963) and specificity (0.935) (González-Palomo et al., 2022). These findings, as well as those of other studies that identified exosomal miRNA biomarkers for DN are summarized in Table 3 and Figure 2 (Eissa et al., 2016a; Eissa et al., 2016b; Delić et al., 2016; Mohan et al., 2016; Xie et al., 2017; Li et al., 2018; Zang et al., 2019).

**TABLE 3:** The role of exosomal components in diabetic nephropathy *in-vivo*. ADSC, adipose-derived mesenchymal stem cell; AEBP1, Adipocyte enhancer binding protein 1; AMBP, ɑ-1-microglobulin/bikunin precursor; AUC, area under the curve; CKD, chronic kidney disease; DN, diabetic nephropathy; ECM, extracellular matrix; Eno1, enolase-1; ER, endoplasmic reticulum; ET1, endothelin-1; HDAC1, histone deacetylase-1; MANF, mesencephalic astrocyte-derived neurotrophic factor; MiR, microRNA; MLL3, upregulated lysine-specific methyltransferase 2C; mTOR, mammalian target of rapamycin; MUP1, major urinary protein 1; NR, not reported; PTEC, proximal tubular epithelial cells; SMAD1, mothers against decapentaplegic homolog 1; TGF-β, transforming growth factor beta; TSP-1, thrombospondin-1; VDAC1, downregulated voltage-dependent anion channel 1.

Primary purpose	Exosomal component	Species	Location	Association with T2DM complication	Potential implications	References
Diagnostic	Xaa-Pro Peptidase, MUP1	Rat	Urine	Increased, decreased	Increase in Xaa-Pro peptidase levels may indicate proteolytic activity (i.e., breakdown of ECM components), leading to renal structural injury seen in DN. Decreased MUP1 suggests a potential dysregulation of this protein, leading to altered renal function and impaired antioxidant defense mechanisms	Raimondo et al. (2013)
Diagnostic	AMBP, MLL3, VDAC1	Human	Urine	Increased, increased, decreased	AMBP is indicative of ongoing renal damage; increased MLL3 and decreased VDAC1 suggest impaired gene expression and mitochondrial dysfunction	Zubiri et al. (2014)
Diagnostic	Eno1	Mouse	Tubular cells (paracrine)	Increased	Exosomal Eno1 from tubular cells may be implicated in the generational of renal fibrosis	Wen et al. (2020)
Diagnostic	AEBP1	Mouse	Serum	Increased	NR	Tao et al. (2021)
Diagnostic	miR-451-5p, miR-16	Rat	Urine	Increased	NR	(Mohan et al., 2016)
Diagnostic	miR-320c, miR-6068, miR-1234-5p, miR-6133, miR-4270, miR-4739, miR-371b-5p, miR-638, miR-572, miR-1227-5p, miR-6126, miR-1915-5pmiR-4778-5p, miR-2861, miR-30d-5p, miR-30e-5p	Human	Urine	Increased (miR-30d-5p and miR-30e-5p are decreased)	miR-320c modifies the TGF-β signaling pathway via TSP-1 targeting and may be a therapeutic target.	Delić et al. (2016)
Diagnostic	miR-15b-5p miR-29c-5p let-7c-5p	Human	Urine	Decreased, decreased, increased	Can be used to predict DN with AUCs of 0.818, 0.774, and 0.818, respectively	Li et al. (2018)
Diagnostic	miR-362-3p, miR-877-3p, miR-150-5p, miR-15a-5p	Human	Urine	Increased, increased, increased, decreased	NR	Xie et al. (2017)
Diagnostic	miR-133b, miR-342, miR-30	Human	Urine	Increased	Potential as preclinical biomarkers as evidenced by changes even in normo-albuminuria patients	Eissa et al. (2016a)
Diagnostic	miR-15b, miR-34a, miR-636	Human	Urine	Increased	NR	Eissa et al. (2016b)
Diagnostic	miR-21-5p, miR-30b-5p	Human	Urine	Increased, decreased	The upregulation of miR-21-5p (and downregulation of miR-30b-5p) was able to distinguish DN from healthy patients, but not from CKD, suggesting that these markers are generally associated with renal impairment but not DN specifically	Zang et al. (2019)
Therapeutic	miR-486	Mouse	Serum	Decreased	miR-486 from ADSC-derived exosomes could protect against DN by regulating podocyte homeostasis via Smad1/mTOR downregulation	Jin et al. (2019)
Therapeutic	miR-125a	Rat	Serum	Decreased	miR-125a from ADSC-derived exosomes provides benefit through downregulation of HDAC1 and subsequently, ET1	Hao et al. (2021)
Therapeutic	miR-92a-1-5p	Mouse	PTECs	Increased	miR-92a-1-5p worsened DN by inducing ER stress via downregulation of calreticulin and MANF.	Tsai et al. (2023)

Beyond their role as biomarkers, exosomes could also be therapeutic for diabetic nephropathy. At the time of the first studies on exosomes as therapies in DN, it was already known that mesenchymal stem cells (i.e., those present in the bone marrow and in mesenchymal tissues; MSCs) function as effective therapeutics for diabetic nephropathy in murine models (Ezquer et al., 2009; Zhang et al., 2013; Abdel Aziz et al., 2014; Ezquer et al., 2015). The mechanism was unknown, however, a leading theory suggested that the therapeutic mechanism of action was via paracrine effects of trophic factors that were secreted by MSCs (Kuroda et al., 2011). Nagaishi et al. attempted to build on this theory, as well as the growing evidence of exosomes from MSCs showing therapeutic potential in studies unrelated to DN, by assessing exosomal effects on diabetic nephropathy (Katsuda et al., 2013; Yu et al., 2014; Nagaishi et al., 2016). Using both streptozotocin-injected mice and high-fat diet mice as diabetic animal models, the group first confirmed the therapeutic effect of MSCs. It tested the therapeutic effect of MSC-conditioned medium. Interestingly, the MSC-conditioned medium mice performed similarly or better than those treated with MSCs as measured by albumin to creatinine ratio, histopathological analysis, and blood glucose levels (Nagaishi et al., 2016). The group further assessed these effects to show that both therapeutics inhibited the infiltration of bone-marrow-derived cells into kidneys, a mechanism that the group had previously shown to be nephrotoxic in the setting of diabetic nephropathy (Yamashita et al., 2012; Nagaishi et al., 2016). Further experiments revealed both MSCs and MSC-condition medium downregulated expression of intercellular adhesion molecule-1, TNF-α, and phosphorylated p38 mitogen-activated protein kinase, which, taken together, suggest a reduction in inflammation. Finally, the improvement of nephrological structural integrity was confirmed by morphological assessment of the proximal tubules, downregulation of transforming growth factor beta (TGF-β) in tubular epithelial cells, indicating less epithelial to mesenchymal transition, and upregulation of zona occludens protein-1, a key marker of tight junction function (Rajasekaran et al., 1996; Fragiadaki and Mason, 2011; Nagaishi et al., 2016). Throughout these experiments, the question remained as to whether the effects seen in the MSC-conditioned medium group were due to exosomes in the medium. Two experiments attempted to address that question. First, the group purified exosomes from MSC-conditioned medium and unilaterally administered them into the subcapsular space of streptozotocin-induced rats. After sacrificing the rats, the two kidneys were compared. The exosome-treated kidneys abated the expansion of renal tubules, atrophy of tubular epithelial cells, and infiltration of inflammatory cells. In addition, downregulation of TGF-β in tubular epithelial cells and upregulation of zona occludens protein-1 were once again noted (Nagaishi et al., 2016). These findings suggest that exosomes are the likely paracrine factor enabling MSC’s therapeutic potential in diabetic nephropathy.

Building on this critical work, other groups identified therapeutic possibilities in exosomes. For example, Jin et al. furthered the therapeutic potential of MSCs (i.e., specifically adipose-derived MSCs) by identifying miR-486 as a likely driver of the benefit seen in *in-vivo* DN mice models. The group identified that miR-486 downregulated renal Smad1 expression, which inhibited mTOR activation, ultimately leading to the promotion of autophagy and the reduction of podocyte apoptosis (Jin et al., 2019). Hao et al. used similar methods to identify miR-125a as a likely driver of the benefit of exosomal therapy from adipose-derived MSCs seen in *in-vivo* DN rat models (Hao et al., 2021). The group suggested that miR-125a bound to histone deacetylase 1 which further downregulated endothelin-1 and provided rescue of glomerular tissue integrity and renal function (Tung et al., 2018; Hao et al., 2021). Tsai et al. harvested proximal tubular epithelial cells and mesangial cells to see if exosomes from the former would drive pathological changes in the latter. Their *in-vitro* exosomal analysis identified miR-92a-1-5p uptake by glomerular mesangial cells and further identified this miRNA to be the driver of myofibroblast transdifferentiation. This suggests miR-92a-1-5p to be involved in the pathogenesis of DN. The group engineered an inhibitor of miR-92-1-5p and showed rescue of nephropathic features both *in-vitro* and in a DN mice model (Tsai et al., 2023).

DN is a complex microvascular complication that significantly impacts the lives of individuals with T2DM. Identifying and characterizing exosomes in DN have contributed to our understanding of the disease pathology and opened up new possibilities for diagnosis and treatment. In particular, several exosomal proteins and miRNAs have emerged as potential biomarkers for DN. Additionally, exosomes derived from MSCs have shown therapeutic potential in preclinical models. The exploration of exosomes in DN represents a promising avenue for the development of non-invasive diagnostic tools and targeted therapeutic interventions. Continued research in this field will further unravel the intricate interplay between exosomes and DN, ultimately leading to improved patient outcomes and a deeper understanding of this condition. A complete list of exosomal involvement in *in-vivo* DN studies is presented in Table 3.

### 5.4 Diabetic peripheral neuropathy

DPN is a debilitating complication of T2DM that affects 50% of individuals with T2DM and estimated to have a worldwide prevalence of 425 million people (Nascimento et al., 2016). DPN is most commonly a symmetric, length-dependent sensorimotor polyneuropathy that manifests as sensory disturbances, neuropathic pain, and impaired motor function (Tesfaye et al., 2010; Feldman et al., 2019). The mechanism by which T2DM induces DPN is not fully understood, but is said to involve microvascular disease, neuroinflammation, and oxidative stress (Feldman et al., 2019). Over the recent years, there has been a growing interest in exploring the role of exosomes as a therapeutic intervention for DPN. Jia et al. were amongst the first to explore the effects of exosomes on DPN and focused on those that were derived from high-glucose (v. non high glucose) stimulated Schwann cells. Their findings identified levels of miR-28, miR-31a, and miR-130a to be higher in exosomes derived from high glucose conditions. The group hypothesized that these exosomes, and likely through the action of these three miRNAs, would accelerate DPN (Jia et al., 2018). *In-vitro* studies confirmed this hypothesis and identified that each of the miRNAs modulate four proteins, an endocytic adaptor protein (NUMB), synaptosome-associated protein 25 (SNAP25), DNA methyltransferase 3a (DNMT3A), and growth-associated protein 43 (GAP43), which are collectively involved in neuroplasticity, axonal growth, and nerve sprouting (Wakamatsu et al., 1999; Johnson, 2003; Frassoni et al., 2005; Nguyen et al., 2007; Feng et al., 2010; Jia et al., 2018). Specifically, miR-28 targeted NUMB, miR-31a targeted SNAP25, and miR-130a targeted both DNMT3A and GAP43; such targeting led to proteomic downregulation. The group’s *in-vivo* work in diabetic mice confirmed an inverse relationship between the three miRNAs and their target proteins in the sciatic nerves. However, interestingly, this correlation was not seen in the dorsal root ganglions, suggesting this exosomal regulation may be focused to distal nerve fibers (Jia et al., 2018).

In an effort to study exosomes from another cell type, Fan et al. conducted a study using MSC-derived exosomes to understand their effects on DPN. The results of the study revealed that treatment with MSC-exosomes significantly improved neurological outcomes in diabetic mice with DPN. The treatment group had a decrease in the threshold for thermal and mechanical stimuli and an increase in the nerve conduction velocity. Histopathological analysis showed enhanced density of blood vessels and increased numbers of intraepidermal nerve fibers, myelin thickness, and axonal diameters in the sciatic nerves. In addition, Western blot analysis demonstrated a shift from proinflammatory M1 macrophage phenotype markers to anti-inflammatory M2 markers. Notably, although these results do suggest a significant improvement, the rescue did not yield return of nerve function to baseline (i.e., non-DPN group) (Fan et al., 2020). Network analysis identified miR-17, miR-23a and miR-125b as exosomal components that targeted genes involved in the TLR4/NF-κB and receptor for advanced glycation end product signaling, which are known to be crucial in DPN pathogenesis (Toth et al., 2007; Lawrence and Fong, 2010; Fan et al., 2020). Interestingly, the group’s subsequent work focused on miR-146 enriched MSC-derived exosomes which showed accelerated *in-vivo* therapy through downregulation of the TLR4/NF-κB pathway (Fan et al., 2021). Identification of this pathway, and the multitude of exosomal components that interact with it, suggest a potential mechanism underlying the exosomes’ therapeutic effects (Lawrence and Fong, 2010; Fan et al., 2020; Fan et al., 2021).

Rather than simply injecting the exosomes into the serum, Singh et al. engineered a novel exosomal delivery mechanism using porous nerve guidance channels that could be implanted at the site of injury. The group also created unique DPN rat models as they introduced crush and cut injuries to the sciatic nerve after a 2-month induction with hyperglycemia. Although the results were collected separately, the findings were largely similar across both injury models. Exosomes derived from bone marrow mesenchymal stromal cells (BMSCs) normalized nerve conduction velocity and compound muscle action potential in DPN rats (i.e., returned function similar to healthy control animals). The treatment also led to the recovery of gastrocnemius muscle mass and morphology. Ultimately, for most of these findings, there was no significant improvement whether the porous nerve guidance channels were used or whether the exosomes were injected intramuscularly of note, the study did not conduct a thorough pathway analysis nor exosomal component analysis (Singh et al., 2021). Instead the authors pointed to the mechanism of action of the exosomes hypothetically, stating that previous studies had identified miR-133b, miR-17/92 cluster, brain-derived neurotrophic growth factor, nerve growth factor, insulin growth factor, and fibroblast growth factor as exosomal components of BMSCs that exhibit positive effects on neural regeneration (Xin et al., 2012; Zhang Y. et al., 2017; Qing et al., 2018; Dong et al., 2019; Singh et al., 2021). As these findings were not verified in the paper, they were omitted from Table 2.

More recently, the work conducted by Kasimu et al. focused on astrocyte-derived exosomes. In their study, the group identified the therapeutic component of these exosomes to be miR-125a-5p (Kasimu et al., 2022). Further pathway analysis found that miR-125a-5p functions by downregulating TRAF6, a key signaling molecule associated with inflammation (Cao et al., 1996; Kasimu et al., 2022) TRAF6 is known to activate NF-кB, which leads to rise of pro-inflammatory cytokines or pathogen-associated molecular patterns, and induce neuropathic pain by activation of JNK/MCP-1 pathway (Lu et al., 2014; Liu et al., 2018). The group’s *in-vivo* diabetic mice model analysis yielded findings that suggested reduced astrocytic activation and downregulation of several inflammatory proteins in the treatment group (i.e., miR-125a-5p tail vein injection) (Kasimu et al., 2022).

Interestingly, all the studies in this pathology have focused on therapeutic interventions (Table 4). In doing so, the studies have revealed important findings regarding the pathogenesis of DPN. As each of the studies has derived their therapeutic exosomes from different cell lines, this confirms previous studies that have mentioned the multitudes of modalities through which DPN develops (Feldman et al., 2019). Further investigation into the cargo and pathways involved in exosomal signaling will enhance our understanding of DPN pathophysiology and create new possibilities for therapeutic interventions.

**TABLE 4:** The role of exosomal components in diabetic peripheral neuropathy *in-vivo*. DPN, diabetic peripheral nephropathy; MiR, microRNA; MSC, mesenchymal stem cell; NF-κB, nuclear factor kappa b; NR, not reported; RAGE, receptor for advanced glycation end product signaling; TLR4, toll-like receptor 4; TRAF6, TNF Receptor Associated Factor 6.

Primary purpose	Exosomal component	Species	Location	Association with T2DM complication	Potential implications	References
Therapeutic	miR-28 miR-31a miR-130a	Mouse	Serum	Increased	Derived from high glucose-stimulated Schwann cells; found to modulate proteins involved neuroplasticity, axonal growth, and nerve sprouting, contributing to the progression of neuropathic symptoms	Jia et al. (2018)
Therapeutic	miR-17 miR-23a miR-125b	Mouse	Serum	Decreased	These miRNAs were derived from MSCs and showed mutli-level regulation of the TLR4/NF-κB and RAGE pathways	Fan et al. (2020)
Therapeutic	miR-146a	Mouse	Serum	Decreased	An accelerated alleviation in neurovascular dysfunction was observed upon loading of miR-146a to MSC exosomes as measured by improvements in nerve conduction velocity thresholds for thermal and mechanical stimuli	Fan et al. (2021)
Therapeutic	miR-125a-5p	Mouse	Serum	Decreased	miR-125a-5p downregulates TRAF6-mediated inflammation, providing clinical benefit as measured by mechanical allodynia and thermal hyperalgesia	Kasimu et al. (2022)

### 5.5 Diabetic retinopathy

DR is the most common complication of T2DM and is a leading cause of visual impairment and blindness worldwide (Cao et al., 2021). It is estimated that over 80% of patients with insulin-dependent T2DM and 50% of insulin-independent diabetic patients will develop DR (Stitt et al., 2016). The pathophysiology of DR is characterized by abnormal angiogenesis, loss of retinal pericytes, increased vascular permeability, and proliferation and migration of retinal endothelial cells (Tarr et al., 2013).

Retinal endothelial cells are largely implicated in the pathogenesis of DR as they are the first cells in the eye to sense and respond to changes in circulating blood glucose (Xing et al., 2017). High glucose conditions have been shown to induce proliferation, migration, and tube formation of human retinal endothelial cells secondary to hyperglycemia-induced retinal inflammation (Cao et al., 2021). Aberrations in pericyte-endothelial cell signaling contribute to abnormal angiogenesis, beginning with disruption of the stable association between pericytes and endothelial cells in normal vasculature. This dissociation leads to increased vascular permeability and initiates endothelial cell proliferation and migration into the surrounding tissue. The subsequent dysfunction of the retinal vasculature causes hypoxia and release of vascular endothelial growth factor (VEGF), which further contributes to endothelial cell proliferation, their dissociation from pericytes, vascular leakage, and neovascularization (Raza et al., 2010). Inflammation of the retinal microvasculature simultaneously accelerates the progression of DR. Finally, aberrant activation of the complement system is thought to be involved in the hyperglycemia-induced retinal inflammation and breakdown of the blood-retina barrier. Plasma exosomes have been implicated in this process and have been shown to increase inflammatory markers in T2DM. Huang et al. demonstrated that exosomes rich in IgG are able to activate the classical complement pathway. T2DM increases the levels of IgG-laden exosomes, resulting in a greater degree of exosome-induced complement activation and downstream retinal vascular damage in diabetic patients. Inhibition of exosome-induced complement activation may offer a potential therapeutic approach to prevent or slow the progression of DR (Huang et al., 2018).

Early work by Beltramo et al. identified the role of EVs in the disruption of pericyte-endothelial cell associations. The group’s seminal *in-vitro* work demonstrated that EVs derived from MSCs are able to enter pericytes, leading to subsequent detachment from endothelial cells, vessel destabilization, increased vascular permeability, and angiogenesis. By culturing MSCs in physiologic and diabetic-like conditions, the group demonstrated that high glucose worsened the retinopathic effects of MSC-derived EVs. Their findings suggested that diabetic-like conditions may contribute to DR pathogenesis through paracrine signaling of EVs (Beltramo et al., 2014). Further work was subsequently conducted by the same group to characterize the relative expression of miRNAs in circulating EVs in T2DM patients with and without retinopathy compared to healthy controls. After confirming that serum EVs isolated from both diabetic groups induced features of DR *in vitro*, the researchers assessed the expression of 754 miRNAs using qRT-PCR and found 3 that were differentially expressed in the DR group compared to the non-DR T2DM and healthy groups (Table 2). Although this early work focused on EVs, their findings identified a role for microvesicles, and specifically miRNA cargo, in the pathogenesis of DR (Mazzeo et al., 2018).

Other studies have similarly focused on miRNA. For example, Kamalden et al. demonstrated that exosomal miR-15a is secreted by pancreatic β-cells via exosomes in response to high-glucose conditions. Levels of exosomal miR-15a in the serum and retina of diabetic patients were significantly elevated, and the degree of elevation was found to correlate with DR severity. The group further conducted *in-vitro* analysis that demonstrated circulating exosomal miR-15a is taken up by retinal cells, inducing oxidative stress and contributing to retinal injury by activating apoptosis (Kamalden et al., 2017). Subsequently, Sangalli et al. aimed to see if exosomal miR-15a could be used as a biomarker to be utilized for screening ahead of the DR’s clinical presentation. The group measured ganglionic cell complex thickness as an indicator for early retinal injury in three groups of human subjects (T2DM, impaired glucose tolerance, and healthy controls). This study was the first to demonstrate that ganglionic cell complex thickness reduction preceded the onset of overt retinopathy or T2DM. Furthermore, this reduction was negatively correlated with the concentration of serum exosomal miR-15a in plasma (Sangalli et al., 2020). Taken together, these experiments suggest that exosomal miR-15a is not only a pathogenic contributor of DR, but it can also be used as an early and correlative marker for DR development and progress, even in patients with preclinical T2DM (Kamalden et al., 2017; Sangalli et al., 2020).

Rather than focus on the utilization of exosomal miRNA as a biomarker, Zhang et al. specifically looked at the therapeutic potential of exosomal miR-126. In a previous study, the group identified miR-126 as a factor that may improve DR by enhancing vascular repair and inhibiting hyperglycemia-induced retinal inflammation (Wang and Yan, 2016). In the present study, the group isolated MSC-derived exosomes from human umbilical cord-derived MSCs and cultured them with high levels of miR-126, leading to overexpression of the miRNA in the exosomes. These exosomes were then either injected intravitreally into diabetic rats or co-cultured with high glucose-treated human retinal endothelial cells. They demonstrated that high glucose levels in both the *in-vitro* and *in-vivo* models increased retinal inflammation (via upregulation of IL-1β, IL-8, and caspase-1) and induced expression of high-mobility group box 1 (HMGB1), a protein that regulates the inflammatory response. The administration of exosomes overexpressing miR-126 was effectively able to reverse the levels of inflammatory markers and inhibit the HMGB1 signaling pathway (Zhang et al., 2019).

Beyond miRNAs, several exosomal lncRNAs have also been implicated in DR. Ye et al. were the first to study the relative expression levels of various plasma exosomal lncRNAs between T2DM patients with and without DR. Using a logistic regression model to analyze the relationships between DR and lncRNAs, the group identified upregulation of lncRNA *DLX6-AS1* in the DR group, suggesting it is likely a risk factor for the pathology. Opposingly, the relative expression of lncRNA *PRINS* and lncRNA *FAM190A-3* was lower in the DR group, suggestive of a role as possible protective factors. After running a univariate analysis, the group identified a multivariate model that includes lncRNA *DLX6-AS1* and lncRNA *PRINS* levels to be a significant predictor for DR with an area under the curve of 0.813 (95%CI: 0.740–0.886) (Ye et al., 2022). Furthermore, Cao et al. identified the possible role of exosomal lncRNA SNHG7 in the pathogenesis of DR by isolating them from MSCs. The group first cultured human retinal microvascular endothelial cells (HRMECs) under high glucose conditions and showed that endothelial damage from high glucose exposure is associated with endothelial-mesenchymal transition, downregulation of lncRNA SNHG7, and upregulation of miR-34a-5p. They then cultured HRMECs with lncRNA SNHG7-overexpressing exosomes, which led to a significant reduction in endothelial-mesenchymal transition and tube formation in HRMECs by downregulating miR-34a-5p expression. These findings suggest that exosomal lncRNA SNHG7 may be therapeutic for DR (Cao et al., 2021).

While the pathophysiology of DR is well understood, exosomes are an emerging field of study in the characterization of DR. They have been shown to have utility in predicting the onset and progression of DR and were recently implicated in the pathogenic mechanisms leading to DR. *In-vivo* studies have confirmed their potential application in changing the trajectory of DR by improving or worsening retinal damage depending on their miRNA or lncRNA content (Table 5). Targeting the exosomal contents to modify their downstream effects may serve as a therapeutic intervention to prevent the development or slow the progression of DR.

**TABLE 5:** The role of exosomal components in diabetic retinopathy *in-vivo*. DR, diabetic retinopathy; lncRNA, long noncoding RNA; MiR, microRNA; MSC, mesenchymal stem cell.

Primary purpose	Exosomal component	Species	Location	Association with T2DM complication	Potential implications	References
Diagnostic	miR-15a	Human	Serum	Increased	Exosomal miR-15a negatively correlates with ganglion cell complex thickness and may be used to monitor pre-clinical retinal damage	Sangalli et al. (2020)
Diagnostic	miR-150-5p, miR-21-3p, miR-30b-5p	Human	Serum	Decreased, increased, increased	Associated with both onset and progression of DR.	Mazzeo et al. (2018)
Therapeutic	miR-126	Rat	Vitreous	Decreased	Administration of MSC-derived exosomes overexpressing miR-126 can reduce hyperglycemia-induced retinal inflammation	Zhang, Wang and Kong (2019)
Diagnostic	lncRNA *DLX6-AS1*, lncRNA *PRINS*, lncRNA *FAM190A-3*	Human	Serum	Increased, decreased, decreased	lncRNA *DLX6-AS1* was identified as a risk factor for DR, and lncRNA *PRINS* and lncRNA *FAM190A-3* were identified as protective factors for DR.	Ye et al. (2022)

### 5.6 Diabetic wound healing

DWH is a well-characterized process that involves cell migration, proliferation, fibrogenesis, angiogenesis, and reepithelialization (Falanga, 2005). In T2DM, this process is significantly impaired, which leads to chronic non-healing ulcers and lower extremity amputation (Rosenberg, 1990). In conjunction with the impaired immune response in T2DM patients, diabetic wounds are a source of potentially life-threatening infections (Brem and Tomic-Canic, 2007). Despite the serious nature of diabetic wounds, treatment options consist primarily of conservative practices, such as dressings, pressure offloading, surgical debridement and revascularization, infection management, and glycemic control, which are supportive rather than curative and often ineffective (Everett and Mathioudakis, 2018). Exosomes offer the opportunity to address the limitations of current practice for DWH management.

The majority of studies on exosomal effects in DWH involve the utilization of MSCs. Zhao et al. investigated such a therapy using exosomes from human adipose-derived MSCs in a diabetic mouse model. The wound healing rate was significantly increased in the exosome-treated group. Further functional assays revealed that these exosomes enhanced diabetic wound healing via a multitude of mechanisms (e.g., reepithelialization, cell proliferation, collagen synthesis, tissue remodeling, skin barrier repair, angiogenesis, and inhibition of apoptosis and inflammation). Finally, the group conducted biochemical pathway analysis that revealed that the exosomes stimulated fibroblast proliferation and migration by negatively regulating MMP1 and MMP3 expression (Zhao et al., 2021).

Li et al. took a different approach to address DWH by first analyzing patient tissue from diabetic foot ulcers (DFUs). After identifying overexpression of miR-152-3p in these tissues, the group was able to subsequently show that exosomes from bone marrow derived MSCs contained lncRNA H19, which was the key inhibitor of miR-152-3p. Further *in-vitro* pathway analysis identified that miR-152-3p mediated its effects by downregulating PTEN, which is vital for preventing apoptosis and inflammation. Finally, the group confirmed exosomal effects by injecting exosomes with overexpression of lncRNA H19 into a DFU mouse model and demonstrating rescue of DFUs. Taken as a whole, this study suggests that MSC-derived exosomal lncRNA H19 stimulates repair of DFUs by promoting fibroblast proliferation and migration and suppressing apoptosis and inflammation via regulation of the lncRNA H19/miR-152-3p/PTEN axis (Li et al., 2020).

Two other groups studied DWH by focusing on the role of MSCs on senescent cells (Bian et al., 2020; Xiao et al., 2021). Bian et al. initially demonstrated that prolonged stimulation with high glucose accelerated senescence of *in-vitro* human dermal fibroblasts (HDFs) while simultaneously suppressing HDF proliferation and migration rates. However, when the group introduced exosomes from decidua-derived MSCs effectively, the effects of high glucose were rescued. Further analysis revealed these exosomes inhibited the generation of reactive oxygen species and protected against HDF senescence by suppressing the expression of advanced glycation end-product receptors. Local application of these exosomes in the wounds of diabetic mice enhanced wound healing suggesting that exosomes from decidua-derived MSCs may be a promising candidate for DWH (Bian et al., 2020). The second group, Xiao et al., focused on the effects of exosomes from adipose tissue derived MSCs and demonstrated that these exosomes were able to rescue senescence of *in-vitro* human umbilical vein endothelial cells induced by both hydrogen peroxide and high glucose. Introduction of these exosomes to a DWH mouse model demonstrated improved wound healing as quantified by scar width and percentage of re-epithelialization. The group went on further to conduct an in-depth genomic analysis of over 1,000 mature miRNAs that ultimately demonstrated the effects of the MSC-exosomes were driven by their miR-146a cargo (Xiao et al., 2021).

Two groups also showed that MSC-derived exosomes improve DWH via their circRNA cargo (Shi et al., 2020; Wang et al., 2023). Leveraging a previous finding that circRNA mmu_circ_0000250 expression is abnormally low in diabetic mice, Shi et al. conducted further *in-vitro* analysis. Their findings revealed that exosomes secreted by mmu_circ_0000250-overexpressing adipose-derived MSCs decreased high glucose-induced endothelial cell apoptosis and increased angiogenesis by promoting autophagy. mmu_circ_0000250 was found to activate autophagy through inhibition of miR-128-3p and subsequent upregulation of sirtuin 1 (SIRT1) (Shi et al., 2020), a protein known to be involved in regulating inflammation, cell migration, and wound healing (Qiang et al., 2017). *In-vivo* application of these exosomes into wounds of diabetic mice improved DWH*,* confirming the *in-vitro* therapeutic findings (Shi et al., 2020). Wang et al. applied similar methodologies and discovered a similar role for exosomal circRNA astrotactin 1 (circ-Astn1) from adipose-derived MSCs. Circ-Astn1 was found to inhibit miR-138-5p and subsequently upregulate SIRT1. Higher concentrations of circ-Astn1 accelerated full-thickness cutaneous wound healing in diabetic mice (Wang et al., 2023). Interestingly, both studies identified a common end-pathway protein for the effects of exosomal circRNA. These findings suggest even more potential therapeutic options (Shi et al., 2020; Wang et al., 2023).

Another therapeutic option that allows for the forgoing of the invasive biopsy required to generate adipose-derived MSCs is urine-derived stem cells (USCs). Chen et al. explored the therapeutic potential of exosomes from human USCs to address DWH. After demonstrating that these exosomes have therapeutic benefit *in-vitro* and *in-vivo*, the group identified that the major mechanism of DWH improvement was through an increase in the rates of reepithelialization, collagen deposition, and vessel formation. Proteomic analysis revealed that a pro-angiogenic protein, deleted in malignant brain tumors 1 (DMBT1), was highly expressed in exosomes from USCs. The group subsequently analyzed the effects of knocking out DMBT1, which yielded return of poor wound healing in both *in-vitro* and *in-vivo* studies. Findings from this study demonstrate that local transplantation of exosomes from USCs is able to accelerate diabetic wound healing in mice and suggests a promising therapeutic strategy in humans (Chen et al., 2018).

Rather than use stem cells, Huang et al. bioengineered exosomes to address wound healing. The group first used patient samples to identify miRNA expression levels in diabetic chronic wounds. After identifying miR-31-5p to be significantly underexpressed in such tissue, they showed that *in-vitro* expression of miR-31-5p promoted angiogenesis, fibrogenesis, and epithelialization. The group subsequently engineered exosomes with miR-31-5p and introduced them to a streptozotocin-induced diabetic rat model. These rats demonstrated a significantly faster rate of wound healing compared to two control groups—those that received non-engineered exosomes and those that received no exosomes. This finding suggests a novel technique towards exosomal therapeutic delivery that bypasses the utilization of stem cells to generate a “cell-free” therapy (Huang et al., 2021).

Taken together, the findings from these studies suggest that the effect of exosomes in DWH is complex and involves a multitude of both RNA and protein cargo. As a result, there are various therapeutic targets that are now available to explore in order to improve DWH management (Table 6). Given that current treatment protocols are largely supportive, implementation of these findings in humans may radically change the future management of this pathology.

**TABLE 6:** The role of exosomal components in diabetic wound healing *in-vivo*. ADSC, adipose-derived mesenchymal stem cell; DMBT1, deleted in malignant brain tumors 1; lncRNA, long noncoding RNA; MiR, microRNA; PTEN, phosphatase and tensin homolog; SIRT1, sirtuin 1; USC, urine-derived stem cell.

Primary purpose	Exosomal component	Species	Location	Association with T2DM complication	Potential implications	References
Therapeutic	miR-31-5p	Rat	Subcutaneous at wound site	Decreased	Engineered miR-31-5p exosomes promote diabetic wound healing by enhancing angiogenesis, fibrogenesis, and reepithelialization	Huang et al. (2021)
Therapeutic	DMBT1	Mouse	Subcutaneous at wound site	Decreased	USC-derived exosomes enriched in DMBT1 promoted angiogenesis and improved wound healing	Chen et al. (2018)
Therapeutic	lncRNA H19	Mouse	Skin at wound site	Decreased	MSC-derived exosomes overexpressing lncRNA H19 stimulate wound healing by suppressing fibroblast apoptosis via impairing miR-152-3p- mediated PTEN inhibition	Li et al. (2020)
Therapeutic	miR-146a	Mouse	Subcutaneous at wound site	Decreased	ADSC-derived exosomes rescued senescence of endothelial cells by downregulating p-Src	Xiao et al. (2021)
Therapeutic	mmu_circ_0000250	Mouse	Subcutaneous at wound site	Decreased	mmu_circ_0000250 in ADSC-derived exosomes enhanced wound healing by miR-128-3p adsorption and subsequent SIRT1 upregulation	Shi et al. (2020)
Therapeutic	circ-Astn1	Mouse	Subcutaneous at wound site	Decreased	circ-Astn1 in ADSC exosomes promoted wound healing via miR-138-5p adsorption and subsequent SIRT1 upregulation	Wang et al. (2023)

## 6 Current clinical trials

As evidenced in Section 5.0, many of the current studies focus on animal models. In order to identify current and past clinical trials, a search on clinicaltrials.gov for studies containing keywords “exosome OR miRNA OR vesicle” in the condition “Type 2 Diabetes” was done in May 2023 and yielded 38 studies. The 38 studies were subdivided into 15 observational studies and 23 were interventional studies. Of the 23 interventional studies, only 15 were truly measuring an exosome or exosomal component. The majority of these studies were using exosomes as an opportunity to quantify the effect of a common diabetic medication or exercise therapy. Of note, only two of these studies were started in 2022 (Trial Registration Numbers: NCT05259449 and NCT05139914), and none were started in 2023. No study was found that was utilizing exosomes as an interventional therapy. A review of the gray literature, however, did identify one open-label phase I/II study that co-cultured mononuclear cells with cord blood-derived multipotent stem cells and was successful in reducing HbA1C levels at 12 weeks and 1 year post-treatment (Zhao et al., 2013). While this study did not directly utilize exosomes as a therapeutic, it has been shown that the therapeutic potential of co-culture with cord blood-derived multipotent stem cells is due to their exosomes (Hu et al., 2020a).

The overall findings reported here suggest that in spite of the overwhelming evidence that exists in mice, rats, and humans about the potential for exosomes as therapeutic opportunities for T2DM, there are currently limited efforts supporting their transition to pharmaceuticals. While it is possible that this could be due to the costs and challenges of exosomal isolation, it is also possible that more time is needed for industry support of these trials given the recency of several of the findings reported in this review (Cheruvanky et al., 2007). As more observational studies are conducted and new, cost-effective, isolation strategies develop, novel therapeutic exosomal trials for T2DM are anticipated (Li et al., 2017).

## 7 Conclusion

Over the last several years, it has become clear that exosomes play an important role in the pathophysiology of T2DM and its complications. The findings presented in this review shed light on the numerous potential diagnostic and therapeutic options that exist within exosomes. As of this review, the large majority of findings are limited to *in-vitro*, *in-vivo*, and observational human studies. In clinical settings (i.e., those that have the capacity to isolate exosomes), serum and urine exosomes can be readily utilized as biomarkers for T2DM and several of its complications. Many of the limitations of exosomes are due to the challenges of their large-scale production and clinical-grade storage (Hu et al., 2020b). As these concerns are resolved, the implementation of randomized control trials will be needed to fully assess the safety and therapeutic potential of exosomes.
